# Cost-Effectiveness of Preoperative Screening and Eradication of *Staphylococcus aureus* Carriage

**DOI:** 10.1371/journal.pone.0014815

**Published:** 2011-05-26

**Authors:** Marjan W. M. Wassenberg, G. Ardine de Wit, Marc J. M. Bonten

**Affiliations:** 1 Department of Medical Microbiology, University Medical Center, Utrecht, The Netherlands; 2 Department of Internal Medicine and Infectious Diseases, University Medical Center, Utrecht, the Netherlands; 3 Julius Center for Health Sciences and Primary Care, University Medical Center, Utrecht, The Netherlands; 4 National Institute of Public Health and the Environment, Bilthoven, The Netherlands; Royal Tropical Institute, The Netherlands

## Abstract

**Background:**

Preoperative screening for nasal *S. aureus* carriage, followed by eradication treatment of identified carriers with nasal mupirocine ointment and chlorhexidine soap was highly effective in preventing deep-seated *S. aureus* infections. It is unknown how cost-effectiveness of this intervention is affected by suboptimal *S. aureus* screening. We determined cost-effectiveness of different preoperative *S. aureus* screening regimes.

**Methods:**

We compared different screening scenarios (ranging from treating all patients without screening to treating only identified *S. aureus* carriers) to the base case scenario without any screening and treatment. Screening and treatment costs as well as costs and mortality due to deep-seated *S. aureus* infection were derived from hospital databases and prospectively collected data, respectively.

**Results:**

As compared to the base case scenario, all scenarios are associated with improved health care outcomes at reduced costs. Treating all patients without screening is most cost-beneficial, saving €7339 per life year gained, as compared to €3330 when only identified carriers are treated. In sensitivity analysis, outcomes are susceptible to the sensitivity of the screening test and the efficacy of treatment. Reductions in these parameters would reduce the cost-effectiveness of scenarios in which treatment is based on screening. When only identified *S. aureus* carriers are treated costs of screening should be less than €6.23 to become the dominant strategy.

**Conclusions:**

Preoperative screening and eradication of *S. aureus* carriage to prevent deep-seated *S. aureus* infections saves both life years and medical costs at the same time, although treating all patients without screening is the dominant strategy, resulting in most health gains and largest savings.

## Introduction

Deep surgical site infections caused by *Staphylococcus aureus* are an important complication of surgical procedures, associated with increased morbidity and mortality and considerable incremental health care costs. It is estimated that around 80% of such infections are caused by strains already colonizing the patient at the time of surgery, most notably in the anterior nares [1]. In a multi-centre double-blind placebo-controlled trial, preoperative screening for nasal *S. aureus* carriage, followed by *S. aureus* eradication treatment of identified carriers with nasal mupirocine ointment and chlorhexidine gluconate soap, all within one week before surgery, was associated with 79% and 55% reductions in deep-seated and superficial *S. aureus* infections, respectively [2]. Yet, screening all patients preoperatively with PCR-based rapid diagnostic tests and providing timely treatment to identified carriers will be an enormous logistical – and costly – challenge. The cost-effectiveness of this intervention has not been determined and it is unknown how this will be affected by suboptimal screening. The aim of this study was, therefore, to quantify costs and effects of this intervention using different screening scenarios.

## Methods

We performed a cost-effectiveness analysis from the societal perspective and restricted our analyses to deep-seated prosthetic joint and deep-seated cardio surgical infections (such as mediastinitis), as the intervention is most relevant for these types of infections. Cost and mortality related to deep surgical site infections were derived from the hospital databases of the UMC Utrecht, one of the participating centres of the recently published multi-centre trial. The UMC Utrecht is a tertiary medical centre with 1012 beds, in which around 200 prosthetic joint implantations and 1,000 cardiopulmonary surgical procedures are performed annually. Infectious complications of these interventions, as well as all costs associated, have been prospectively monitored from 2001 on by the department of hospital hygiene and infection control. From this database we calculated costs for patients readmitted because of a postoperative surgical site infection, which included the number of hospital admission days, surgical procedures, and diagnostic procedures (mainly radiological and microbiological) (Table 1). We intentionally restricted cost estimates to the costs associated with readmission for deep surgical infections in order to not to include other non-associated costs. Although we might have missed episodes that were readmitted to other hospitals, we believe this number to be low and such cases were unlikely to influence the calculated cost per episode. We assessed hospital mortality of patients with deep-seated *S. aureus* prosthetic joint and cardio surgical infections and quantified the average number of expected life years at the time of death using life tables from Statistics Netherlands (Table 2). We did not adjust life expectancy for comorbidities. Therefore, life expectancies are overestimated, which implies that the calculated savings per life year are rather conservative. Institutional review board approval was not required as monitoring postoperative and other hospital associated infections is part of the regular infection control program conducted by the department of hospital hygiene and infection control.

**Table 1:** Direct health care costs of patients readmitted because of a postoperative surgical site infection between 2001 and 2010.

	Department	
	Cardio surgical	Orthopedic
	(n = 28)	(n = 25)
Age, mean ± SD, years	58 ± 16	55 ± 15
Male sex (%)	19 (68)	13 (52)
Surgical interventions, no. (%)		
coronary artery bypass grafting	20 (71)	0
heart valve replacement	3 (11)	0
total knee or hip prosthesis	0	7 (28)
shoulder surgery	0	3 (12)
spondylodesis	0	3 (12)
other fracture surgery	0	6 (24)
other	5 (18)	6 (24)
Length of hospital stay, total days (median)	529 (17)	1597 (48)
Total cost of health care, euros (mean/patient)		
surgical procedures	31,605 (1580)^a^	71,695 (2987)^b^
laboratory investigations	40,149 (1434)	31,781 (1271)
radiological investigations	9068 (363)^c^	5071 (211)^b^
hospitalization days	150,157 (5363)	499,728 (19,989)
other	3716 (133)	17,705 (708)
total costs readmission^d^	234,695 (8382)	625,980 (25,039)

a Missing information on 8 patients.

b Missing information on 1 patient.

c Missing information on 3 patients.

d Costs of antibiotic treatment were not included.

**Table 2:** Life expectancy of patients with deep-seated postoperative *S. aureus* infections.

Characteristic	n = 37^a^
Age, mean ± SD, years	66 ± 17
Male sex (%)	19 (51)
Hospital department (%)	
cardio surgical	23 (62)
orthopedic	14 (28)
Postoperative *S. aureus* infections, no. (%)	
mediastinitis	18 (49)
prosthetic joint infection	14 (38)
other deep-seated infection	5 (14)
In-hospital deaths, no. (%)	9 (24)
Expected survival, mean, years	
survivors	19.83
survivors, discounted	9.54
non survivors	15.83
non survivors, discounted	9.29

a 17 patients (46%) are included in estimate of the cost of a postoperative surgical site infection.

Parameters used in the model are listed in Table 3. The incidence of deep-seated *S. aureus* infections in *S. aureus* carriers was 4.4% and was assumed to be three to six times lower (1.5% to 0.7%) among non-carriers [3]–[5]. In order to be conservative in our estimates of cost-effectiveness, we have used a six times lower incidence in our calculations. The relative risk of deep-seated *S. aureus* infections after mupirocin-chlorhexidine treatment was 0.21 compared to placebo [2]. Sensitivity and specificity of the rapid diagnostic test was considered 0.97 and 0.99, respectively [2], [6].

**Table 3:** Parameters used in cost-effectiveness analysis.

Parameter	Value	Reference
No. of patients colonized with *S. aureus*, %	18.5	[2]
Incidence of deep-seated *S. aureus* infections among *S. aureus* carriers, %	4.4	[2]
Incidence of deep-seated *S. aureus* infections among non-carriers, %	0.7-1.5	[2]
Cost of screening, €	63.90	[7]
Sensitivity of screening test	0.97	[2]-[5]
Specificity of screening test	0.99	[6]
Cost of treatment, €	15.94	[8]
Relative risk of deep-seated *S. aureus* infections due to intervention, %	0.21	[2]
Cost of deep-seated surgical site infection, €	17,820	UMC Utrecht
Hospital mortality among patients with deep-seated *S. aureus* infection, %	24	UMC Utrecht
Life expectancy of non-survivors of deep-seated *S. aureus* infection, years	15.83	UMC Utrecht
Life expectancy of non-survivors of deep-seated *S. aureus* infection, discounted, years	9.29	UMC Utrecht

The calculated costs of the screening strategy included material and labour costs (real-time PCR assay and conventional culture costs as determined by the Dutch Healthcare Authority) [7]. We assumed 5 minutes extra working time for nurses (the average nurse wage was valued at €26.45 per hour based on gross salary including taxes and social premiums). Costs of mupirocin ointment 2% and chlorhexidine gluconate soap (40 mg per millilitre) for 5 days were calculated according to the pharmaceutical reference pricing system [8]. The reference year for cost computations was 2009. Costs related to postoperative surgical site infection have been adapted to 2009 using the consumer price index as determined by Statistics Netherlands. Effects (life years gained) were discounted at 3%, as recommended in the U.S.A. [9]; costs were not discounted, as all costs are made in one year. Costs were expressed in Euros (1 Euro  = 1.43 US dollars, December 2009).

Two scenarios were compared to the base case scenario, in which none of the patients will be screened or treated for *S. aureus* carriage. In scenario 1, it is aimed to screen all patients and only identified *S. aureus* carriers receive eradication treatment, as in the study by Bode et al [2]. Yet, preoperative screening and allocating treatment within one week before surgery may not be achievable in all patients, for which estimates of 15% have been reported [10], [11]. Therefore, in scenario 2 not only identified *S. aureus* carriers but also patients that could not be screened receive eradication treatment. In each scenario, proportions of patients screened were varied from 0% (making scenario 1 identical to base case, and treating all patients in scenario 2) to 100% (making scenario 1 and 2 identical). For each of the scenarios total number of *S. aureus* infections prevented, costs per infection prevented and costs per life year gained were determined. In sensitivity analyses, we explored which uncertain variables in the model were of most influence on the cost-effectiveness of screening strategies.

## Results

**Figure 1:**
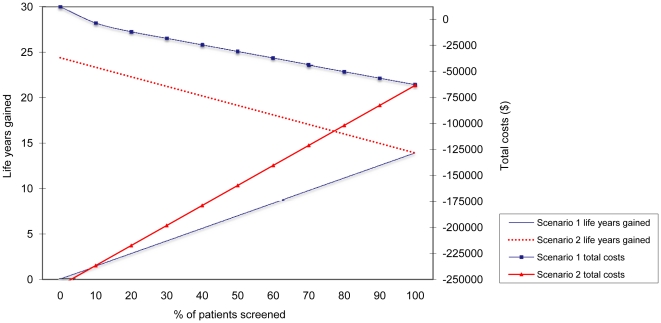
Effect of suboptimal adherence to preoperative *S. aureus* screening on total costs and life years gained (discounted) per 1000 patients for scenario 1 (only identified *S. aureus* carriers are treated) and scenario 2 (identified *S. aureus* carriers and non-screened patients are treated). Note the negative costs on the y-axis representing cost-savings.

Without screening and treatment (base case) there will be 14 deep-seated postoperative *S. aureus* infections with 3 patients succumbing from the infection per 1000 procedures, incidence 1.4% (Table 4). Despite large cost differences between scenarios 1 and 2, both scenarios are associated with improved health outcomes and cost savings, compared to the base case scenario without screening and treatment (Figure 1). Yet, the scenario with all patients treated without screening (scenario 2 with 0% of patients screened) clearly is most cost-effective with highest health gains (24 discounted life years) and highest cost savings (€178,970) per 1000 patients. These positive results are explained by the avoidance of screening costs and from the higher efficacy of the intervention, as carriers are not missed and non-carriers, though with a lower infection risk, are also treated.

**Table 4:** Cost-analysis of different strategies per 1000 patients undergoing prosthetic joint or cardiopulmonary surgery.

Parameter	Base case Scenario	Scenario 1 (identified *S. aureus* carries are treated: 100% screened)	Scenario 1 (identified *S. aureus* carries are treated: 85% screened)	Scenario 2 (identified *S. aureus* carries and non-screened patients are treated: 85% screened)	Scenario 2 (identified *S. aureus* carries and non-screened patients are treated: 0% screened)
No. of patients screened	0	1000	850	850	0
No. of patients treated	0	185	153	303	1000
No. of deep-seated *S. aureus* infection	14	7	9	7	3
No. of deaths due to deep-seated *S.*					
*aureus* infection	3	2	2	2	0.7
No. of life years gained, discounted	-31	14	12	15	24
Cost per infection prevented, €	NA	10,395	10,703	8518	1457
Cost per life year gained, €	7993	-3330	-3192	-4172	-7339
Total costs saved due to infection					
prevention, €	-246,722	47,746	37,737	64,583	178,970

**Figure 2:**
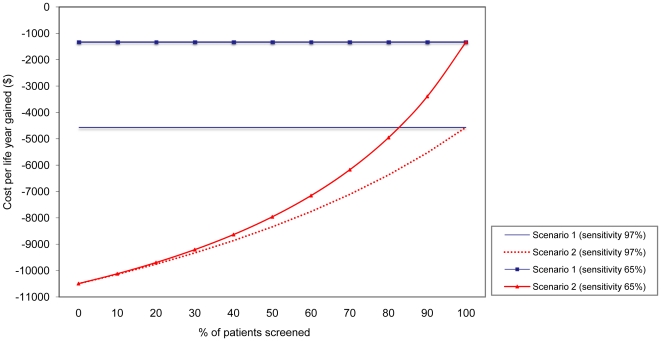
Effect of suboptimal adherence to preoperative *S. aureus* screening and test sensitivity on cost per life year gained (discounted) per 1000 patients for different screening scenarios. Scenario 1 is treatment of *S. aureus* carriers identified by screening; scenario 2 is treatment of *S. aureus* carriers identified by screening *plus* treatment of all patients that were not screened. In scenario 1 the life years gained and costs increase, or decrease, at a constant rate resulting in an invariable cost per life years gained. Note the negative costs on the y-axis representing cost-savings.

If test sensitivity of the PCR-based screening test is 65%, as was reported for the test used in the multi-center trial [6], the intervention would be less beneficial (and save €930 per life year gained) in scenario 1 (Figure 2). In scenario 2 (non-screened patients will all be treated) the effect of test sensitivity on costs per life year gained decreases with less patients being screened.

**Figure 3:**
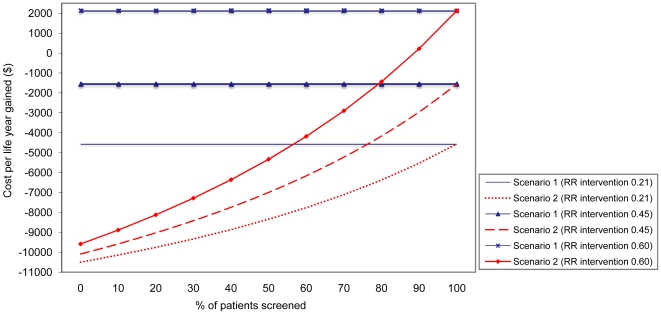
Effect of suboptimal adherence to preoperative *S. aureus* screening and treatment effectiveness on cost per life year gained (discounted) per 1000 patients for different screening scenarios. Scenario 1 is treatment of *S. aureus* carriers identified by screening; scenario 2 is treatment of *S. aureus* carriers identified by screening *plus* treatment of all patients that were not screened. In scenario 1 the life years gained and costs increase, or decrease, at a constant rate resulting in an invariable cost per life years gained. Note the negative costs on the y-axis representing cost-savings.

We have assumed that the relative risk of deep-seated *S. aureus* infections after eradication treatment was 0.21 compared to placebo. However, if the effect of the intervention is lower, health outcomes and cost savings are less (Figure 3), and when the relative risk with eradication is less than, respectively, 0.53 and 0.62 for scenario 1 and 2, the intervention is no longer cost-saving.

If the proportion of identified *S. aureus* carriers is higher than 18.5% [1], [11], the cost savings will increase for all scenarios. The largest increase occurs in scenario 1, in which the cost savings per life year gained will increase from €3330 (with 18.5% carriage) to €5039 with 30% carriage, which is still lower than the €7339 per life year gained when all patients are treated without screening.

When only identified *S. aureus* carriers receive eradication treatment (scenario 1) costs of screening should be less than €6.23 to become the dominant strategy, resulting in more cost-savings than scenario 2 with 0% screening. In scenario 2, when all non-screened patients also receive treatment, the break-even point of the screening costs will decrease with fewer patients being screened. For instance, with 85% of patients being screened treating only identified *S. aureus* carriers will become the dominant strategy if the costs of screening become less than €50.27 per patient.

## Discussion

Although screening for *S. aureus* carriage followed by short-term eradication therapy among identified carriers is associated with both improved health outcomes and reduced health care costs, treating all patients without previous screening appears to result in more infections prevented, more life years gained and higher savings. The benefits in life years gained and costs saved must be balanced against the risks for selection of mupirocin and/or chlorhexidine resistance by using these agents in patients not colonized with *S. aureus*.

The current analysis was motivated by our own experiences with implementing preoperative screening for *S. aureus* carriage in our hospital. A large - and increasing - number of patients is admitted just hours before surgery, with the last out-patient clinic visit frequently weeks before. In such patients preoperative screening and starting treatment is difficult to realize. Furthermore, although the recent clinical trial reported PCR test sensitivity of 97% [2], the sensitivity as reported in literature is much lower (65% to 82%) [6], [12]. Although negative screening results have been associated with lower levels of colonization density [6], possibly reflecting lower infection risks [3], [13], patients potentially benefiting from eradication therapy will be missed in a screen and treat strategy. These aspects did not affect the multi-centre trial as only *S. aureus* carriers, as identified with PCR-testing, were eligible. Numbers of patients that were not screened were not available from the Bode publication, but in our hospital we estimate that more than 50% of the surgical patients were not screened, due to the before-mentioned reasons. Moreover, the 18.5% incidence of *S. aureus* carriage in the multi-centre study was lower than previously estimated, probably because of low test sensitivity.

Strengths of our analysis include the fact that both costs and mortality due to deep-seated postoperative *S. aureus* infections were based on detailed prospectively determined data. Yet, we still had to make assumptions on several parameter values, which deserve comments. For instance, the *S. aureus* infection rate among non-carriers was estimated to be three to six times lower than among carriers based on published data [2], [5]. We have used a six times lower incidence in our calculations, which is a conservative estimate as a higher incidence of infection would result in increased savings. Also, the intervention was considered equally effective in *S. aureus* carriers and non-carriers. Since the overwhelming majority of *S. aureus* infections are from endogenous origin, this assumption seems not unlikely. Yet, a lower efficacy of treatment in non-identified carriers would make any strategy that includes screening less cost-effective. Furthermore, we assumed that all patients with deep-seated infections that died in our hospital (i.e., 24%, all cardiosurgical patients) succumbed because of the infection. Others though have estimated attributable mortality due to serious *S. aureus* infections to range from 7–20% [14], [15]. With lower mortality rates the number of life years gained would become lower, but the relative differences between the different scenarios would not change. We also limited our analyses to deep-seated infections, as we had most accurate cost data for this category of infection and the effect of the intervention was most pronounced for these infections in the recently published trial [2]. Real-life cost-effectiveness is assumed to be more beneficial than reported in this paper, as we disregard the effects of screen and treat strategies on superficial infections. Only direct health care costs were included in our analysis. As the average age of patients dying from nosocomial *S. aureus* infections in our hospital was almost 70, inclusion of indirect costs associated with productivity losses is not thought to have a large influence on our estimates.

Widespread use of mupirocine and chlorhexidine might increase the risk of resistance of *S. aureus*, as resistance for both agents has been reported. However, overall resistance rates for both agents are still low, despite widespread use in the last decades. Some *S. aureus* strains carry the plasmid-born *qac*A/B genes which code for multidrug efflux pumps and increase minimum bactericidal concentrations (MBCs) to chlorhexidine [16]. Although presence of these genes have been associated with reduced efficacy of a chlorhexidine-based decolonization strategy to prevent MRSA transmission in a British ICU [17], MBCs usually remain below concentrations to treat patients and the clinical significance of these *qac*A/B genes remains unclear [18]. Yet, whether treatment in the absence of *S. aureus* colonization increases the risk of resistance to mupirocine and chlorhexidine (for instance through selection of plasmid-born resistance in coagulase-negative staphylococci) is unknown. Yet, based on the cost-effectiveness of decolonization to prevent deep-seated postoperative *S. aureus* infections, we conclude that the benefits of treating non-detected or non-screened patients outweigh the future risks of reduced effectiveness due to widespread resistance to antiseptics and mupirocine. Surveillance cultures will be necessary for early detection of emerging resistance.
